# COVID-19 and Pregnancy: Have We Gotten through the Darkest Hour?

**DOI:** 10.3390/jpm12121987

**Published:** 2022-12-01

**Authors:** Alessandro Favilli, Antonio Simone Laganà, Vito Chiantera, Stefano Uccella, Sandro Gerli, Simone Garzon

**Affiliations:** 1 Unit of Obstetrics and Gynecology, Department of Medicine and Surgery, University of Perugia, 06123 Perugia, Italy; 2 Unit of Gynecologic Oncology, ARNAS “Civico—Di Cristina—Benfratelli”, Department of Health Promotion, Mother and Child Care, Internal Medicine and Medical Specialties (PROMISE), University of Palermo, 90127 Palermo, Italy; 3 Unit of Obstetrics and Gynecology—Department of Surgery, Dentistry, Pediatrics, and Gynecology, AOUI Verona—University of Verona Piazzale A. Stefani 1, 37126 Verona, Italy

On December 2019, a new Severe Acute Respiratory Syndrome Coronavirus (SARS-CoV-2) was isolated and identified in Wuhan (China). Within just a few months, the global health system was shocked by a pandemic caused by this highly contagious virus, which rapidly spread across the world, causing severe acute respiratory syndrome (COVID-19) [1,2]. It has been estimated by official sources that the number of lives lost to the pandemic was 5.9 million worldwide, but in a recent new analysis, a number three times higher has been speculated, close to 18 million [3]. This novel scenario led to significant changes in healthcare policy organization, even in the field of gynecology and obstetrics [4,5,6,7]. Pregnant women’s healthcare in the context of COVID-19 represented a new challenge for midwives and obstetricians due to the lack of scientific evidence that could help in the management of such complex clinical cases [8,9,10,11,12,13]. Indeed, pregnancy itself represents a particular condition that is characterized by a natural suppression of the immune system and higher susceptibility to infectious diseases [14].

During the COVID-19 pandemic, new evidence was progressively raised from clinical practice. For instance, it has been demonstrated that SARS-CoV-2 infection in pregnant women is the same as in the general population [15], although COVID-19 disease increases morbidity in pregnant women and fetuses, placing pregnancy as a high-risk condition [16,17,18]. Indeed, pregnant people with COVID-19 are more likely to become seriously ill, which can lead to a high risk for a composite outcome of maternal mortality or serious morbidity from obstetric complications during and after pregnancy, as well as higher risk for cesarean delivery, postpartum hemorrhage, hypertensive disorders of pregnancy, and preterm birth [19]. Nevertheless, the pathway to obtaining adequate knowledge about how to manage pregnancies affected by COVID-19 was not immune to obstacles. Indeed, pregnancy and breastfeeding status are generally considered a limited condition to investigate and/or to test new pharmacological and clinical insights. Probably due to unfounded concerns about the risk to the fetus, the “protection by exclusion” issue has excluded pregnant women from potentially beneficial treatments, making it extremely difficult to assess the safety and efficacy of drugs in pregnancy [20,21,22].

In critical cases, infected pregnant women represented a clinical conundrum with heavy ethical and deontological repercussions. The great dilemma that clinicians had to face was how to assess the optimal delivery mode based on obstetric conditions and COVID-19 severity. Nowadays, it has become clear that the management of COVID-19 in pregnancy should be handled by a multidisciplinary team, including obstetricians, physicians, anesthetists, and intensivists who could be called to make a decision regarding timing, place, and mode of delivery. Despite the fact that mild clinical manifestations can benefit from standard treatment such as fever symptomatic therapy [23], more complex clinical cases should be addressed to reference centers in which critical care, such as respiratory support with oxygen, ventilation in a prone position with or without intubation, and up to recourse to extracorporeal membrane oxygenation (ECMO), can be offered [20].

Since the beginning of the pandemic, several pieces of evidence have progressively emerged, which have significantly modified the standard care and management of both non-pregnant [24,25] and pregnant women affected by COVID-19. Initially, breastfeeding was considered as a risk of infection for newborns from COVID-19-infected mothers, and cesarean section and strict isolation of the neonate were deliberated as ways to keep newborns healthy and safe. Breastfeeding has now been demonstrated to be safe and may in fact help babies gain immunity that can protect them from COVID-19 [26]. Moreover, it has been speculated that the presence of anti-SARS-CoV-2 antibodies in breast milk may express a possible specific protective effect on the newborn infant after both maternal infection and vaccination [27]. New observational studies are ongoing to evaluate the immune responses generated by COVID-19 in pregnant or postpartum people (including breastfeeding women) who received the vaccine.

The advent of the vaccine represented a breakthrough in the battle against COVID-19. The development of vaccines and the global start of vaccination campaigns signaled a new era, saving countless lives and reducing the severe effects of the disease in the world population [28]. Nevertheless, the emergency use and lack of exhaustive clinical information regarding effects on pregnancy and fetus have caused fears and skepticism, which initially limited the spread of vaccination and its benefits. Today, in absence of contraindications, the main scientific societies agree that vaccinations should be offered to all pregnant and breastfeeding women after they have been adequately informed of the benefits, especially in case of the presence of comorbidities such as obesity, diabetes, heart disease, as well as lung disease [27]. However, it has been estimated that the prevalence of COVID-19 vaccine acceptance in pregnant women is 53.46%, which is much lower than the general COVID-19 vaccination rate [28]. Waiting to test the effectiveness of vaccines currently available against potential new variants, these data give strength regarding the importance of proceeding with informative campaigns which may reassure and inform pregnant women about the benefits of COVID-19 vaccination during pregnancy. 

Much new evidence has emerged in these years, and there is more still coming out. Despite this, several issues must be still clarified which can offer new weapons to win the battle against COVID-19. One of the matters still debated and investigated is represented by the potential vertical transmission of infection from the mother to the fetus and related newborn outcomes. To date, the vertical transmission of SARS-CoV-2 during pregnancy remains controversial. Until now, only a few newborns have been reported as positive for COVID-19 after birth [29]. It is still unknown if the newborns’ infection was obtained before, during, or after birth [30]. Only new data from well-designed clinical trials will shed light on these unresolved issues.

The scientific community went through the darkness of COVID-19 following the light coming from what was already known about previous experience with other highly pathogenic coronaviruses, namely severe acute respiratory syndrome (SARS) and Middle East respiratory syndrome (MERS). [31]. Since December 2019, relevant knowledge about the pathogenesis and management of SARS-CoV-2 has been collected, and at present, clinicians can face COVID-19 with new and effective instruments backed by solid scientific evidence. Although the COVID-19 pandemic seems to have surpassed its “darkest hour”, the scientific community cannot afford to lower its guard against the possibility of new waves of resurgence, which could jeopardize the effectiveness of our current knowledge about healthcare for pregnant people and fetuses. Sharing data among the scientific community to improve the quality of care in maternal health, as well as supporting healthcare providers in infection management, should be of priority importance [32]. Researchers must remain alert to offer the most timely and effective evidence-based care to pregnant women with COVID-19 disease, in addition to in the possible case of the development of new variants. Reducing and preventing maternal and neonatal morbidity related to COVID-19 must remain a global objective.
